# Switch On the Night: Policies for Smarter Lighting

**DOI:** 10.1289/ehp.117-a28

**Published:** 2009-01

**Authors:** Luz Claudio

It was an August afternoon in 2003 when the lights went out on Broadway, and for that matter, throughout most of the Northeast, Midwest, and Ontario—a power blackout left 50 million customers in the dark overnight. Despite complaints about the inconveniences, the stranded commuters, and the food spoilage in restaurants and markets, many city dwellers were also awed; as evening came on, they gazed upward, and between the dark skyscrapers they could see something amazing—the starry night sky. *The New York Times* reported spontaneous stargazing gatherings in the usually electrified cities of the Northeast.

On ordinary nights with electric power, the bright sky glow surrounding cities at night can extend some 150 miles from population centers, thus obscuring the view of the stars for most of the population. In a December 2006 survey, the nonprofit Campaign to Protect Rural England and the British Astronomical Association’s Campaign for Dark Skies asked people across the United Kingdom to count how many stars they could see in the constellation Orion. More than half the 2,000 respondents could see fewer than 10 of Orion’s stars, whereas astronomers say around 250 should be visible to the naked eye on a moonless night.

Artificial nighttime lighting does more than just obscure the stars. A growing number of studies are linking light pollution to a variety of human and environmental health effects [see “Missing the Dark: Health Effects of Light Pollution,” p. A20 this issue]. Moreover, polluting light is often wasted light involving unnecessary energy costs and carbon dioxide (CO_2_) emissions. In response to these concerns—as well as a desire for better night sky viewing—many cities are exploring ways to regulate artificial lighting and implement smarter lighting strategies.

## Blinded by the Light

Artificial lighting does not necessarily produce light pollution. *Light pollution* is the term for artificial light that is excessive or intrudes where it is not wanted. Well-designed lighting, on the other hand, sends light only where it is needed without scattering it elsewhere. Experts in the field agree that light pollution can be easily controlled with well-designed lighting and simple measures such as turning off indoor and outdoor lights when not in use.

Defining and measuring light pollution is not yet quite as easy, however. In the November/December 2004 issue of the *IMSA* [International Municipal Signal Association] *Journal*, researcher Michele McColgan of the Lighting Research Center at Rensselaer Polytechnic Institute wrote, “The problem facing authorities is twofold: how to identify the balance between useful lighting and light pollution; and how to quantify lighting objectively to see if this balance is being met.” McColgan and colleagues have developed a so-called shoebox metric that may make it easier to quantify and thus regulate light pollution. The “shoebox” moniker refers to the rectangular area described by the vertical and horizontal planes surrounding a site. The researchers propose that architects and planners consider the amount of light leaving the shoebox along each plane as a way of characterizing a site’s lighting impact. Policy makers could also regulate how much light is allowed to exit each plane.

Contrary to popular belief, bright lighting can actually hinder visibility rather than improve it, according to the nonprofit International Dark-Sky Association (IDA), which provides guidance on selecting good outdoor lighting and creating local lighting ordinances. For example, light that shines horizontally (as from headlights and some styles of light fixtures) produces glare that can momentarily obstruct visibility, especially on roadways and on wet nights.

There is also some debate over whether brighter outdoor lighting actually improves security. *Preventing Crime: What Works, What Doesn’t, What’s Promising*, a 1996 report submitted to the U.S. Congress by the National Institute of Justice and the Department of Criminology and Criminal Justice at the University of Maryland, concluded that “lighting is effective in some places, ineffective in others, and counter productive in still other circumstances” in deterring crime. The authors hypothesized that high levels of artificial lighting can conceivably increase the likelihood of crime when one considers that offenders need the light to detect potential targets and low-risk situations. The report also proposed that bright outdoor lighting may make pedestrians feel safer but also make them more visible to offenders. However, in the 27 February 2007 issue of *JAMA*, Dana Loomis and colleagues from the University of North Carolina at Chapel Hill demonstrated that bright lighting both indoors and outdoors at business establishments lessened the likelihood of workers being murdered on the job.

IDA contends that it is poorly designed lighting that compromises safety. For instance, glare can create safety issues around buildings by causing very sharp shadows and temporarily blinding passersby to would-be assailants. In its information sheet “Security Lighting: Let’s Have Real Security, Not Just Bad Lighting,” the group makes several recommendations for effective security lighting, such as the use of “full cutoff” fixtures, meaning the bulb is recessed within an opaque lampshade or shield that focuses the light downward, which prevents glare.

Sometimes less light can be an effective deterrent to crime. On 25 November 2008 the *Colchester* (U.K.) *Gazette* reported that towns in Essex County, United Kingdom, had seen a reduction in crime during an 18-month pilot project in which most residential streetlights were turned off between midnight and 5:00 a.m. In Maldon, the number of recorded offenses fell by 14% during the hours the streetlights were off, and offenses overall fell by 12.6%. The Essex County Council, which originally intended the measure as a way to curb energy-related CO_2_ emissions, is currently considering whether to implement the program countywide.

## A Basis in Astronomy

Advocacy for mitigating light pollution has been especially strong in the astronomy community. Some of the first proponents of reducing outdoor light at night were astronomers, both amateurs with backyard telescopes and professionals whose work is impeded by light in the environment.

IDA, in conjunction with Lowell Observatory and the U.S. Naval Observatory, has worked with the city of Flagstaff, Arizona, and surrounding Coconino County to issue ordinances requiring proper shielding of outdoor lights. Flagstaff was already a pioneer in light pollution awareness; the city council passed the first ever lighting ordinance in April 1958, banning advertising searchlights that interfered with work at nearby Lowell Observatory. Flagstaff’s commitment to the preservation of dark skies through proactive lighting codes and public education led to the city being designated the first International Dark-Sky City by IDA in 2001.

Truly dark skies have become somewhat of a rarity and, for many people, a natural treasure worth preserving. In 1999 the National Park Service formed the Night Sky Team to address increasing public concern about light pollution. “We are charged with protecting the scenery and habitats of our national parks, and that includes the night sky,” says Chad Moore, Night Sky Program manager. The Night Sky Team is developing instruments and methods to help measure light pollution, such as a portable field instrument that can quantify natural and artificial sky lighting and quickly image the entire sky in high resolution. The team is also creating an inventory of present night sky conditions in national parks where the viewing is clearest, to set a baseline against which light pollution can be assessed.

For several national parks, darkness has become a main attraction. For example, Natural Bridges National Monument in Utah is known as a prime place to view the Milky Way. For Bryce Canyon National Park, also in Utah, and the Chaco Culture National Historical Park in New Mexico, stargazing is the number one attraction, drawing 15,000 to 30,000 visitors per year.

Stargazing is not the only pastime that depends on the dark to draw tourists. Puerto Rico is famous for its bays where bioluminescent *Pyrodinium bahamense* dinoflagellates set the water aglow at night, and swimming and kayaking in the dark bay waters releases swirls of blue–green light. Although pollution from boat fuel and pesticide runoff threatens the dinoflagellates themselves, according to a 9 June 2008 report on National Public Radio’s *Morning Edition*, light pollution affects the tourist value of the bays by greatly reducing the visual impact of the bioluminescence. In part to protect these and other sensitive ecosystems on the island and thereby also protect the country’s ecotourism trade, the government of Puerto Rico signed into law the Program for the Control and Prevention of Light Pollution in August 2008.

## Preserving the Rhythm of the Night

Although astronomers were the first to express concern about the effects of artificial nighttime lighting, Travis Longcore, a research associate professor at the University of Southern California Center for Sustainable Cities, says concern about the effects of light pollution on wildlife and plants has been a more recent phenomenon. Today, much of the impetus for addressing light pollution comes from its disruptive ecologic effects.

In Florida’s Sarasota County, the problem of light pollution was particularly acute because this area of the Florida coast is a significant sea turtle nesting zone, where a number of threatened and endangered species lay their eggs every season from May through October. Adult females and turtle hatchlings alike are affected by artificial nighttime light, which interferes with their ability to navigate to and from nesting areas.

Several methods for saving the turtles were tried, including caging and artificial hatcheries, but none of these efforts worked as well as mandatory lighting codes. In 1997, the county passed the Marine Turtle Protection Code. Among other measures, the code prohibits floodlights, uplights, and spotlights that are directly visible from the beach or that indirectly illuminate the beach, and requires the use of motion detectors for exterior security lights.

As of January 2008, 27 counties and 58 municipalities in Florida had enacted lighting ordinances aimed at protecting sea turtles. “There are successes that would show that the [statewide] program has been effective in raising public awareness regarding lights and sea turtles,” says Jean Higgins, an environmental specialist with the Florida Wildlife Conservation Commission Imperiled Species Management Section. “All in all, the program works hard to see improvements in areas. But it is definitely at times an uphill battle—development continues along Florida’s coastline, and making sure that everyone is aware of the issues at hand as well as equipped with the correct information is difficult.”

Migrating birds also are particularly affected by bright and blinking lights, which confuse them and cause them to crash into buildings and communication towers as they fly their nighttime migration patterns. In the city of Toronto alone, the nonprofit Fatal Light Awareness Program has documented more than 42,000 bird collisions since 1993. Many of the birds are endangered or threatened species.

Toronto issued its Bird-Friendly Development Guidelines in 2007 to provide developers, building managers, architects, and urban planners with design-based strategies to reduce artificial light and glare from buildings. The authors of the guidelines emphasize the need to reduce upward-pointed lighting and turn off unnecessary indoor and outdoor lights at night, especially during the migratory season. For businesses where people work at night, the authors recommend the use of task lighting at individual work stations and drawing the blinds or curtains to minimize the amount of light leaving the building.

## Targeting Energy Consumption

Various estimates posit that lighting accounts for about 8–9% of the electricity used in the United States. In unpublished calculations, the IDA Technical Committee recently estimated that 17.4 billion kilowatt-hours of electricity—requiring 186.3 trillion BTU of energy to produce at power plants—is wasted each year. This waste arises, for instance, from lighting that is directed upward, illuminating nothing but sky or that is left on when not needed. According to the Energy Information Administration, it takes more than 9 million tons of coal or 32 million barrels of oil to produce that amount of energy. This translates into annual CO_2_ emissions of nearly 1 ton or 2.5 tons, depending on the fuel, according to EPA conversion factors.

On a March evening in 2007, in an effort to raise public awareness about energy waste and climate change, the World Wildlife Fund (WWF) held the first Earth Hour in Sydney, Australia. The WWF estimated that turning off nonessential lights for 1 hour in the evening would result in a 5% reduction in energy consumption, but later reported the event resulted in a 10% drop in energy use in the city.

An added effect of lowering the lights during Earth Hour was increased public awareness about light pollution. “The symbolic gesture of turning out the lights for Earth Hour gave people a voice on this issue and increased people’s awareness about the unnecessary lighting we have in our homes and that adorn buildings around the world,” says Dan Forman, public relations manager for the WWF. Forman says an estimated 50 million people in more than 35 countries participated in the second annual Earth Hour. The next Earth Hour is scheduled for 8:30 p.m. local time on 28 March 2009. The WWF hopes 1 billion people in 1,000 cities worldwide will participate.

The Dark Sky Society, a Long Island, New York–based advocacy group, has published guidance that clearly illustrates the types of light fixtures that best protect against light pollution, which often are the most energy-efficient choices as well. Most of the recommended fixtures have full cut-off designs. Susan Harder, founding member of the Dark Sky Society, says many towns on Long Island attach this guidance to every building permit, and local planning departments can also consult the society’s *Guidelines for Good Exterior Lighting Plans* when developing new sites. “We have been very successful with the ordinances we have promoted because, unlike other codes such as noise, once a light has been changed, it will not be a repeat offender,” says Harder.

Fernando Abruna, an architect and past president of the U.S. Green Building Council Caribbean Chapter, says many of the remedies to light pollution are easily applied. “There are very simple architectural changes that reduce energy consumption and make a huge difference in the light pollution problem. Most times, it is just as simple as changing a light bulb or fixture,” he says.

Lighting designers are looking at other ways to simultaneously curb light pollution and save energy. The Civil Twilight Design Collective, a design group based in San Francisco, has conceptualized a “lunar-resonant” streetlight. Current streetlights are outfitted with photosensitive cells that prompt them to turn on as darkness approaches. The lunar-resonant streetlights would have a similar but much more sensitive cell that would respond to ambient moonlight, allowing the lights to dim and brighten according to the phases of the moon. The designers estimate the lights could save as much as 80–90% of the energy used in streetlighting. In 2007 the project won *Metropolis Magazine*’s Next Generation® Design Prize.

## The National Level

To date the drive to address light pollution has been spearheaded by individual communities, but the idea of a national response to the problem is now being broached. For instance, as part of its Leadership in Energy and Environmental Design program for sustainable design, the U.S. Green Building Council recommends the use of electric fixtures that reduce energy consumption and light pollution. In summer 2008, IDA sponsored briefings before the U.S. Senate and the House of Representatives at which Longcore and other experts presented data on the energy, human health, and environmental health ramifications of light pollution.

On 30 July 2008, 11 members of the U.S. Congress signed a bipartisan letter to Environmental Protection Agency (EPA) administrator Stephen L. Johnson urging the agency to take action on the issue of light pollution. The congressional members proposed four actions the EPA should take to address light pollution: codify an official definition of light pollution that captures the detrimental effects of unchecked nighttime lighting; incorporate research on light pollution in future programs; support education about light pollution in the agency’s education, outreach, and grants programs; and expand the agency’s Energy Star publications and standards to include discussion of appropriate outdoor light fixtures.

Energy Star is a joint effort of the Department of Energy (DOE) and the EPA, begun in 1992 to promote more efficient energy use in homes and businesses. On 20 August 2008 the DOE released a draft proposal to add full shielding and other design specificiations for LED (light-emitting diode) streetlights to the Energy Star standards. The goal is to increase energy efficiency, but the benefit is multifold: “Good design saves money, saves energy, and also just happens to save the night time sky,” says IDA managing director Pete Strasser. (In the particular case of LED streetlights, however, he notes that the whiter light they emit is of concern for both luminance levels and visual response, as well as for potential health concerns pertaining to circadian rhythm disruption.)

It remains to be seen whether regulation of light pollution will take effect as part of a broad national effort or continue to be addressed by local communities. Regardless, an increasing body of research suggests that exposure to artificial light at night disrupts a number of biologic functions in humans, especially those influenced by cyclical hormones such as melatonin. Says Steven Lockley, an assistant professor of medicine in the Division of Sleep Medicine at Harvard Medical School, “We are in desperate need of controlled studies to measure the health impact of street lighting and other exposure to artificial light at night.”

## Using the Night Sky to Foster Scientific Literacy

January kicks off the International Year of Astronomy 2009, an effort by the International Astronomical Union and UNESCO to help citizens around the world rediscover both the nighttime and daytime sky, and thereby better appreciate how basic science affects our daily life. As part of the commemorations, the StarPals International Young Astronomers Network will link students via the Internet to remotely operated research-grade telescopes in New Mexico, Israel, and Australia with which they will be able to view and record images of deep space. StarPals has also launched the StarParks Program for Girl and Boy Scouts, which establishes small oases within a community that are kept dark for night sky viewing.

“StarParks can offer a place to view the International Space Station fly-bys or to even observe a meteor shower, comet, or lunar eclipse,” says IDA member Audrey Fischer, who created and leads StarPals. With these projects, Fischer says, children are not just reading about what it is like to be a scientist; they actually become young scientists, “then yearn to learn more.” She adds, “The average child in America is unaware of what a starry sky is meant to be.”

There are no studies that show whether being able to see the stars influences scientific curiosity in children, but Nadine G. Barlow, an associate professor in the Department of Physics and Astronomy at Northern Arizona University in Flagstaff, believes that it does, based on her own experience. “Here in Flagstaff, because of the lighting restrictions, we can actually see our Milky Way galaxy from the middle of campus,” she says. “Students get more interested in the topic when they can see things with their own eyes.”

## Figures and Tables

**Figure:**
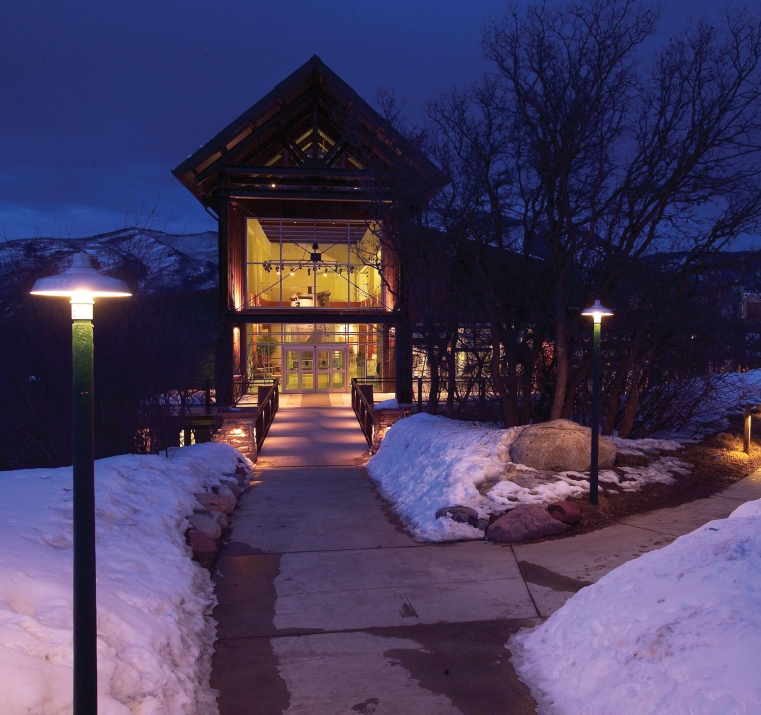
The Aspen Recreation Center in Aspen, Colorado, won the 2004 IDA Lighting Design Award. The award recognizes lighting designs that are free of glare, use optimal levels of light, are energy-efficient, and provide pleasant ambience with minimal obtrusive light and contribution to sky glow. Lights that are fully shielded and well spaced keep light where it is directed, minimizing light pollution.

